# Association Between Changes in Paravalvular Leakage After Transcatheter Aortic Valve Implantation and Serum Lactate Dehydrogenase

**DOI:** 10.14740/cr2307

**Published:** 2026-10-24

**Authors:** Akihiro Ikuta, Gakuto Bando, Satoki Oka, Shunsuke Matsushita, Naoya Inoue, Shingo Hirao, Mitsuru Abe, Kazushige Kadota, Tatsuhiko Komiya, Yasushi Fuku

**Affiliations:** a Department of Cardiovascular Medicine, Kurashiki Central Hospital, Kurashiki, Japan; b Department of Cardiovascular Surgery, Kurashiki Central Hospital, Kurashiki, Japan

**Keywords:** Transcatheter aortic valve implantation, Paravalvular leakage, Serum lactate dehydrogenase

## Abstract

**Background:**

The association between changes in paravalvular leakage (PVL) and serum lactate dehydrogenase (LDH) levels in patients undergoing transcatheter aortic valve implantation (TAVI) is unexplored. We aimed to investigate the short-term changes in PVL after TAVI and their association with LDH elevation.

**Methods:**

A total of 550 consecutive patients underwent TAVI from 2020 to 2024. Of these, 313 receiving SAPIEN 3 (S3) or SAPIEN 3 Ultra RESILIA (S3UR) valves were included in this study. Patients were categorized into two groups based on whether their PVL severity worsened by at least one grade from immediately after the procedure to hospital discharge.

**Results:**

PVL deterioration was observed in 16.3% of patients. The incidence of moderate or greater LDH elevation was significantly higher in the PVL deterioration group than in the non-PVL deterioration group at 7 days (17.6% vs. 8.4%; P = 0.04) and 1 month (29.4% vs. 8.8%; P < 0.01) after the procedure. The area under the receiver operating characteristic (ROC) curve for predicting PVL deterioration using the sizing index (SI) was 0.84, with an optimal cut-off value of 96.5% (odds ratio (OR): 13.6; 95% confidence interval (CI): 6.55–31.1; P < 0.01). Multivariable analysis showed that an aortic valve calcium score ≥ 1,435, use of the S3UR valve, and an SI < 96.5 were independently associated with PVL deterioration.

**Conclusion:**

Early PVL deterioration after TAVI with a balloon-expandable valve may be associated with LDH elevation. These findings highlight the importance of selecting a prosthetic valve with an appropriate sizing ratio to minimize PVL deterioration after TAVI.

## Introduction

Transcatheter aortic valve implantation (TAVI) has become an established therapeutic option for patients with severe aortic stenosis [1]. The widespread adoption of TAVI has been driven in part by advancements in device technology, which have led to improved transvalvular pressure gradients and a reduced incidence of mild or greater paravalvular leakage (PVL) [2]. Nevertheless, the incidence of mild or greater PVL after TAVI remains relatively higher than that after surgical aortic valve replacement, and the severity of PVL may worsen over time due to factors including prosthetic valve recoil after deployment [3, 4]. In addition, previous studies have suggested an association between PVL after TAVI and serum lactate dehydrogenase (LDH) levels; however, it remains poorly explored [5]. While mid-term changes in PVL have been documented, there are limited data on short-term changes and the contributing factors are not well understood [2]. The objective of this study was to investigate the short-term changes in PVL after TAVI and their association with serum LDH levels.

## Materials and Methods

This was a retrospective, single-center observational study. The study population consisted of 550 consecutive patients with severe aortic stenosis who underwent TAVI at our institution from January 2020 to October 2024. After excluding patients who received self-expandable valves (n = 222), experienced unsuccessful valve deployment (n = 5), or underwent valve-in-valve procedures (n = 10), 313 patients who received balloon-expandable SAPIEN 3 (S3) or SAPIEN 3 Ultra RESILIA (S3UR) valves (Edwards Lifesciences) were included in the final analysis (Fig. 1). The choice of valve type was based solely on the implantation period, with S3 used from January 2020 to March 2023 and S3UR adopted from April 2023 onward following institutional transition.

**Figure 1:**
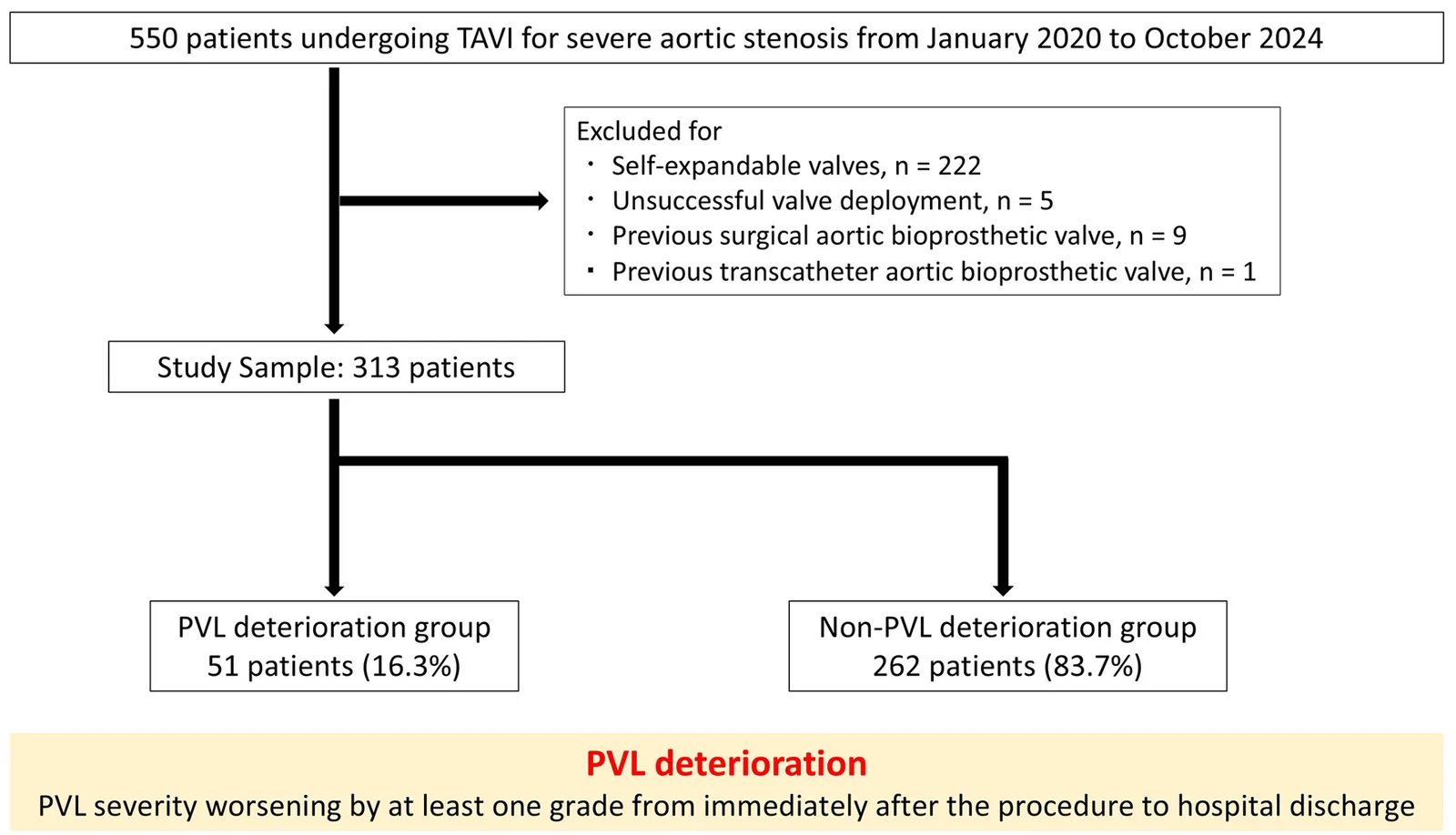
Study flow chart. PVL: paravalvular leakage; TAVI: transcatheter aortic valve implantation.

Clinical and laboratory data were extracted from medical records, including values obtained during hospitalization and from outpatient follow-up. Transthoracic echocardiography (TTE) was performed at baseline, immediately after the procedure, before hospital discharge, and at 1 month after TAVI. Serial PVL assessment was performed using TTE at each time point. TTE was either performed at our clinical echocardiographic laboratory or in cooperation with the patient’s treating cardiologists. PVL was independently assessed based on Valve Academic Research Consortium-3 (VARC-3) criteria and graded using a six-category scheme: none/trace, mild, mild-to-moderate, moderate, moderate-to-severe, or severe [6]. PVL deterioration was defined as an increase of at least one grade in PVL severity from immediately after the procedure to hospital discharge. Patients with PVL deterioration were categorized into the PVL deterioration group and those without into the non-PVL deterioration group. Data on the possible occurrence and/or causes of adverse events were obtained from the medical records or treating hospital team by interview at a planned hospital visit or by telephone interview and questionnaire. The effective orifice area was calculated after the procedure using the continuity equation and indexed to the body surface area (BSA) to derive the indexed effective orifice area. Prosthesis–patient mismatch (PPM) was defined as severe (≤ 0.65 cm^2^/m^2^) or moderate (0.66–0.85 cm^2^/m^2^), based on the indexed effective orifice area [7].

This study was conducted in accordance with the Declaration of Helsinki and the guidelines for epidemiological studies issued by the Japanese Ministry of Health, Labor and Welfare. Informed consent was obtained from all patients for the procedure and use of data for research purposes. Therefore, it was not possible to involve patients or the public in the design, conduct, reporting, or dissemination of the research. The study protocol was approved by the Institutional Ethics Committee of the Kurashiki Central Hospital (Approval Number: 4648).

### Laboratory tests and severity of LDH levels

Serum levels of LDH, alkaline phosphatase (ALP), alanine aminotransferase (ALT), aspartate aminotransferase (AST), hemoglobin, creatine phosphokinase (CPK), total bilirubin (T-Bil), uric acid (UA), and creatinine were measured the day before TAVI, on post-procedural days 1, 3, 7, and at 1-month follow-up. Aortic valve calcification was quantified using the Agatston method and expressed in arbitrary units (AUs) [8]. The degree of LDH elevation was evaluated using modified Horstkotte’s criteria, which are defined as follows: LDH < 220 U/L (none), LDH: 220–399 U/L (mild), LDH: 400–799 U/L (moderate), LDH: 800–999 U/L (severely compensated), and LDH ≥ 1,000 U/L (decompensated) [9]. Clinically relevant hemolysis was defined as hemolysis requiring red blood cell transfusion and/or re-intervention.

### TAVI procedures

TAVI was performed under either general anesthesia with endotracheal intubation or local anesthesia with conscious sedation. All patients received pre-procedural antiplatelet therapy. Valve size selection was based on multi-detector computed tomography (CT)-derived annular measurements rather than balloon sizing. The sizing index (SI) was retrospectively calculated by incorporating the actual balloon inflation volume as follows:

$$\text{SI (\%)}=\frac{\text{Volume-adjusted valve area}}{\text{Measured annular area}}\times \text{100}$$

The volume-adjusted valve area was calculated using the method reported by Shimura et al, which uses valve size and inflation volume [10]. The annular area and perimeter were measured in a short-axis plane during mid-systole. Vascular access was obtained via transfemoral, transapical, subclavian, brachiocephalic, transcarotid, or transaortic routes, depending on anatomical suitability. Vasopressors were administered as needed to ensure adequate coronary perfusion during periods of hemodynamic instability.

### Statistical analysis

Categorical variables were expressed as counts (percentages) and compared using the chi-square test. Continuous variables are presented as mean ± standard deviation (SD) or median (interquartile range, IQR), according to their distribution, and categorical variables as numbers and percentages. Continuous variables were compared using Student’s *t*-test or the Wilcoxon rank-sum test, as appropriate. Categorical variables were compared using the chi-square test or Fisher’s exact test, as appropriate. The Kaplan–Meier plot was used to describe the cumulative mortality between categories. Comparisons of continuous variables were made between the PVL deterioration and non-PVL deterioration groups. Variables with a P value ≤ 0.10 in univariate analyses were included in multivariate logistic regression models to identify independent predictors of PVL deterioration. The number of variables included in the multivariable model was restricted to avoid model overfitting. Multicollinearity was assessed using variance inflation factors. The predictive performance of the SI was evaluated using the receiver operating characteristic (ROC) curve analysis, and the area under the curve (AUC) was used to assess discriminative ability. To assess whether PVL deterioration was independently associated with subsequent LDH elevation, multivariable linear regression analysis was performed with the LDH level at 1 month as the dependent variable, adjusting for baseline LDH, age, sex, and baseline serum creatinine. As a sensitivity analysis, the percent change in LDH from baseline to 1 month was analyzed as the dependent variable, with adjustment for age, sex, and baseline serum creatinine. All statistical analyses were performed using JMP version 9.0 (SAS Institute). A two-tailed P value < 0.05 was considered statistically significant.

## Results

### Baseline patient characteristics and procedural outcomes

Baseline patient characteristics and procedural outcomes are summarized in Tables 1, 2, and Supplementary Material 1 (cr.elmerpub.com), respectively. The mean age was 84.3 ± 5.0 years, the mean Society of Thoracic Surgeons risk score was 7.4 ± 5.5, and the median length of hospital stay was 7.8 days (IQR: 6.7–8.9 days). At hospital discharge, 16.3% of patients exhibited PVL deterioration (PVL group), while the remaining 83.7% did not (non-PVL deterioration group).

**Table 1:** Baseline Patient Characteristics

	Overall	PVL deterioration group	Non-PVL deterioration group	P value
Number of patients	313	51	262	
Age, years	84.3 ± 5.0	84.2 ± 5.3	84.4 ± 4.9	0.81
Age ≥ 85 years	151 (48.2)	23 (45.1)	128 (48.9)	0.62
Men	130 (41.5)	20 (39.2)	110 (42.0)	0.71
BSA, m^2^	1.53 ± 0.18	1.55 ± 0.18	1.52 ± 0.18	0.39
NYHA			-	0.08
I/II	242 (77.3)	44 (86.3)	198 (75.6)	
III/IV	71 (22.7)	7 (13.7)	64 (24.3)	
Society of Thoracic Surgeons risk score	7.4 ± 5.5	7.0 ± 4.3	7.4 ± 5.7	0.59
Hypertension	254 (81.2)	47 (92.2)	207 (79.0)	0.02
Diabetes mellitus	87 (27.8)	16 (31.4)	71 (27.1)	0.54
Dyslipidemia	122 (39.0)	21 (41.2)	101 (38.6)	0.73
Heart failure hospitalization	153 (48.9)	24 (47.1)	129 (49.2)	0.78
Echocardiographic parameters				
LVEF, %	58.8 ± 12.5	61.0 ± 11.3	58.4 ± 12.7	0.18
Mean aortic gradient, mm Hg	45.8 ± 13.0	45.9 ± 14.2	45.8 ± 12.8	0.95
Aortic valve area, cm^2^	0.74 ± 0.22	0.77 ± 0.17	0.73 ± 0.23	0.31
AR ≥ mild	177 (56.6)	36 (70.6)	141 (53.8)	0.02
AR ≥ moderate	41 (13.1)	7 (13.7)	34 (13.0)	0.89
MR ≥ moderate	61 (19.5)	6 (11.8)	55 (21.0)	0.11
Computed tomography parameters				
Annulus area, mm^2^	452 ± 82	453 ± 78	451 ± 82	0.88
Annulus perimeter, mm	75.6 ± 6.9	74.1 ± 6.4	75.9 ± 7.0	0.09
LVOT calc ≥ moderate	17 (5.4)	6 (11.8)	11 (4.2)	0.048
Calcium score	1,480 ± 308	1,769 ± 360	1,425 ± 263	< 0.01
LCC calcium score	451 ± 107	505 ± 127	404 ± 95	< 0.01
NCC calcium score	605 ± 121	707 ± 147	585 ± 105	< 0.01
RCC calcium score	455 ± 120	557 ± 147	435 ± 103	< 0.01
Bicuspid valve	17 (5.4)	6 (11.8)	11 (4.2)	0.048

Values are mean ± standard deviation or n (%), unless otherwise specified. AR: aortic regurgitation; BSA: body surface area; LVEF: left ventricular ejection fraction; LCC: left-coronary cusp; LVOT: left ventricular outflow tract; MR: mitral valve regurgitation; NCC: non-coronary cusp; NYHA: New York Heart Association classification; PVL: paravalvular leakage; RCC: right-coronary cusp.

**Table 2:** Procedural Outcomes

	Overall	PVL deterioration group	Non-PVL deterioration group	P value
Number of patients	313	51	262	
Fluoroscopy time, min	17.9 ± 7.5	16.8 ± 6.0	18.1 ± 7.7	0.25
General anesthesia	251 (80.2)	45 (88.2)	206 (78.6)	0.1
Anesthesia time, min	118 ± 50	107 ± 31	120 ± 53	0.09
Contrast volume, mL	39 ± 20	35 ± 20	40 ± 20	0.12
Blood transfusion	17 (5.4)	3 (5.9)	14 (5.3)	0.87
Transfemoral	270 (86.3)	48 (94.1)	222 (84.7)	0.052
Valve				< 0.01
SAPIEN 3	233 (74.4)	24 (47.1)	209 (79.8)	
SAPIEN 3 Ultra RESILIA	80 (25.6)	27 (52.9)	53 (20.2)	
Valve size				< 0.01
20 mm	16 (5.1)	3 (5.9)	13 (5.0)	
23 mm	170 (54.3)	34 (66.7)	136 (51.9)	
26 mm	103 (32.9)	11 (21.6)	92 (35.1)	
29 mm	24 (7.7)	3 (5.9)	21 (8.0)	
Under volume	204 (65.2)	36 (70.6)	168 (64.1)	0.37
Sizing index	97.8 ± 5.5	93.2 ± 3.9	98.6 ± 5.4	< 0.01
Balloon pre-dilation	155 (49.4)	19 (37.3)	136 (51.7)	0.06
Balloon post-dilation	30 (9.6)	1 (2.0)	29 (11.1)	0.02
Conversion to open heart surgery	0 (0)	0 (0)	0 (0)	
Vascular complications				
Minor	1 (0.3)	0 (0)	1 (0.4)	0.55
Major	11 (3.5)	2 (3.9)	9 (3.4)	0.86
Pacemaker implantation	9 (3.0)	1 (2.2)	8 (3.2)	0.7
Hemodynamic support needed	0 (0)	0 (0)	0 (0)	
Paravalvular leakage				
≥ mild	43 (13.7)	2 (3.9)	41 (15.7)	0.01
≥ moderate	2 (0.6)	0 (0)	2 (0.8)	0.4
Postprocedural mean aortic gradient, mm Hg	11.3 ± 3.8	10.9 ± 3.9	11.4 ± 3.8	0.37
Length of hospital stay, days	7.8 (6.7–8.9)	7.6 (6.5–8.4)	7.9 (6.8–9.2)	0.67
EOA, cm^2^	1.92 ± 0.56	1.94 ± 0.61	1.92 ± 0.55	0.88
Indexed EOA, cm^2^/m^2^	1.27 ± 0.36	1.26 ± 0.39	1.27 ± 0.35	0.84
PPM				
≥ moderate	20 (6.4)	4 (7.8)	16 (6.1)	0.65
≥ severe	1 (0.3)	1 (2.0)	0 (0)	0.55
Clinically relevant hemolysis	2 (0.6)	2 (3.9)	0 (0)	< 0.01

Values are mean ± SD or the median (IQR: 25th–75th percentile), or n (%), unless otherwise specified. EOA: effective orifice area; PPM: prosthesis–patient mismatch; PVL: paravalvular leakage; SD: standard deviation; IQR: interquartile range.

### Changes in laboratory parameters after TAVI

Serial changes in laboratory parameters and corresponding percentage changes are presented in Supplementary Materials 2 and 3 (cr.elmerpub.com). The day before TAVI, UA levels were significantly higher in the PVL deterioration group than in the non-PVL deterioration group, whereas no other laboratory parameters differed significantly between the two groups.

The rate of increase in T-Bil was significantly higher in the PVL deterioration group than in the non-PVL deterioration group on post-procedural days 1 and 3; however, this difference did not persist thereafter. In contrast, LDH levels exhibited a greater and more persistent increase in the PVL deterioration group, starting on post-procedural day 1 and lasting for 1 month. The temporal changes in LDH levels from baseline to 1 month are illustrated in Figure 2.

**Figure 2:**
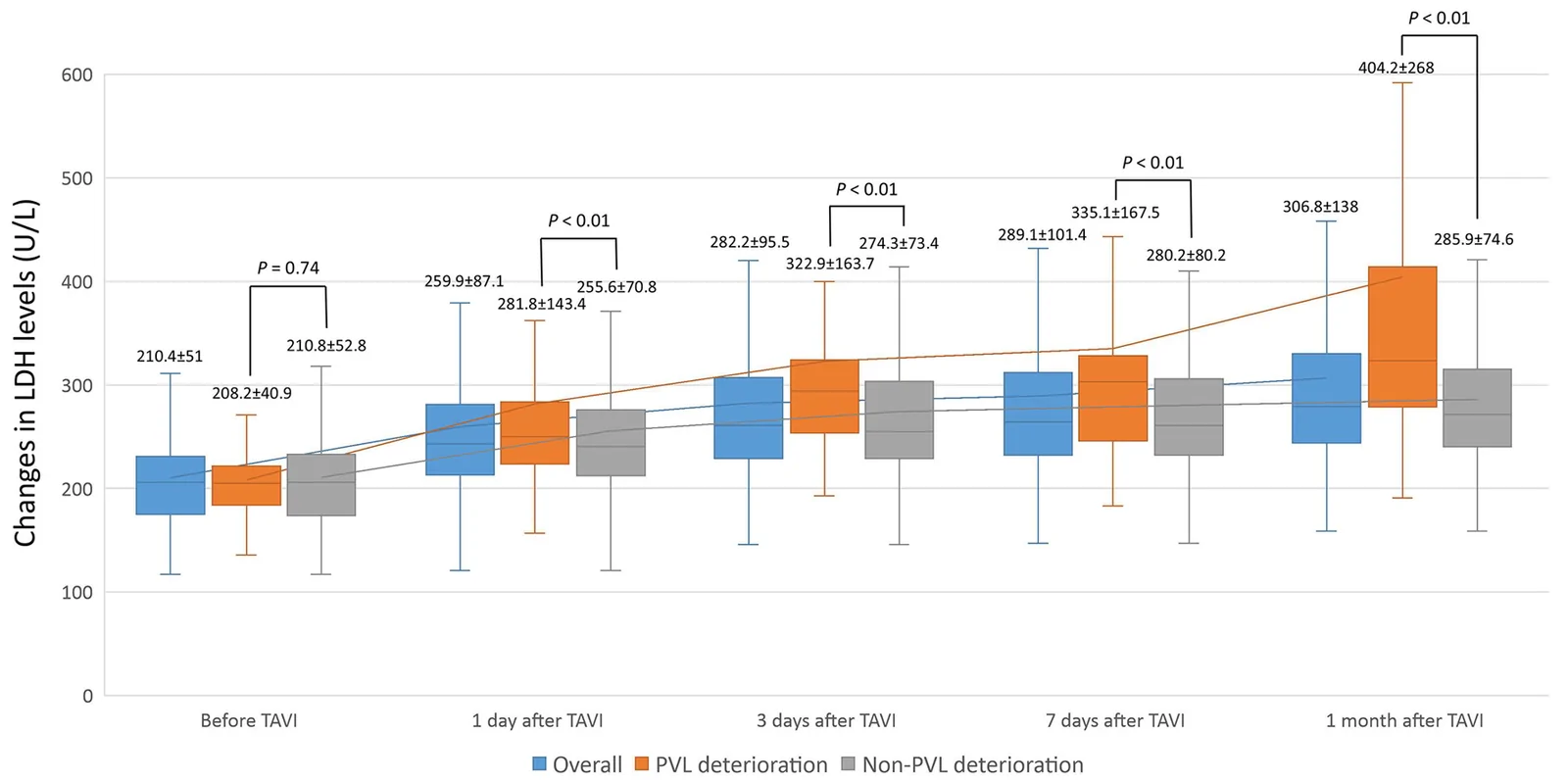
Changes in LDH levels before TAVI and 1 day, 3 days, 7 days, and 1 month after TAVI. LDH: lactate dehydrogenase; PVL: paravalvular leakage; TAVI: transcatheter aortic valve implantation.

In contrast to LDH, CPK levels did not significantly differ between the PVL deterioration and non-PVL deterioration groups at baseline or at 1 day, 3 days, 7 days, and 1 month after TAVI. Similarly, the percent changes in CPK from baseline did not significantly differ between the two groups at any time point; Supplementary Materials 2 and 3 (cr.elmerpub.com).

In the multivariable linear regression analysis, PVL deterioration remained independently associated with a higher LDH level at 1 month after adjustment for baseline LDH, age, sex, and baseline serum creatinine (adjusted β coefficient: 124.1 U/L; 95% CI: 47.1–201.1; P = 0.002); Supplementary Material 4 (cr.elmerpub.com). This association remained consistent in the sensitivity analysis using the percent change in LDH from baseline to 1 month as the dependent variable (adjusted β coefficient: 56.3 percentage points; 95% CI: 29.7–82.8; P < 0.001).

### Severity of serum LDH levels after TAVI

The distribution of severity of serum LDH elevation after TAVI is shown in Figure 3. Moderate or greater serum LDH elevation was observed in 6.1% of patients on post-procedural day 1, 7.3% on day 3, 9.9% on day 7, and 12.1% at 1 month. Notably, the incidence of moderate or greater serum LDH elevation was significantly higher in the PVL deterioration group than in the non-PVL deterioration group on day 7 (17.6% vs. 8.4%; P = 0.04) and at 1 month (29.4% vs. 8.8%; P < 0.01).

**Figure 3:**
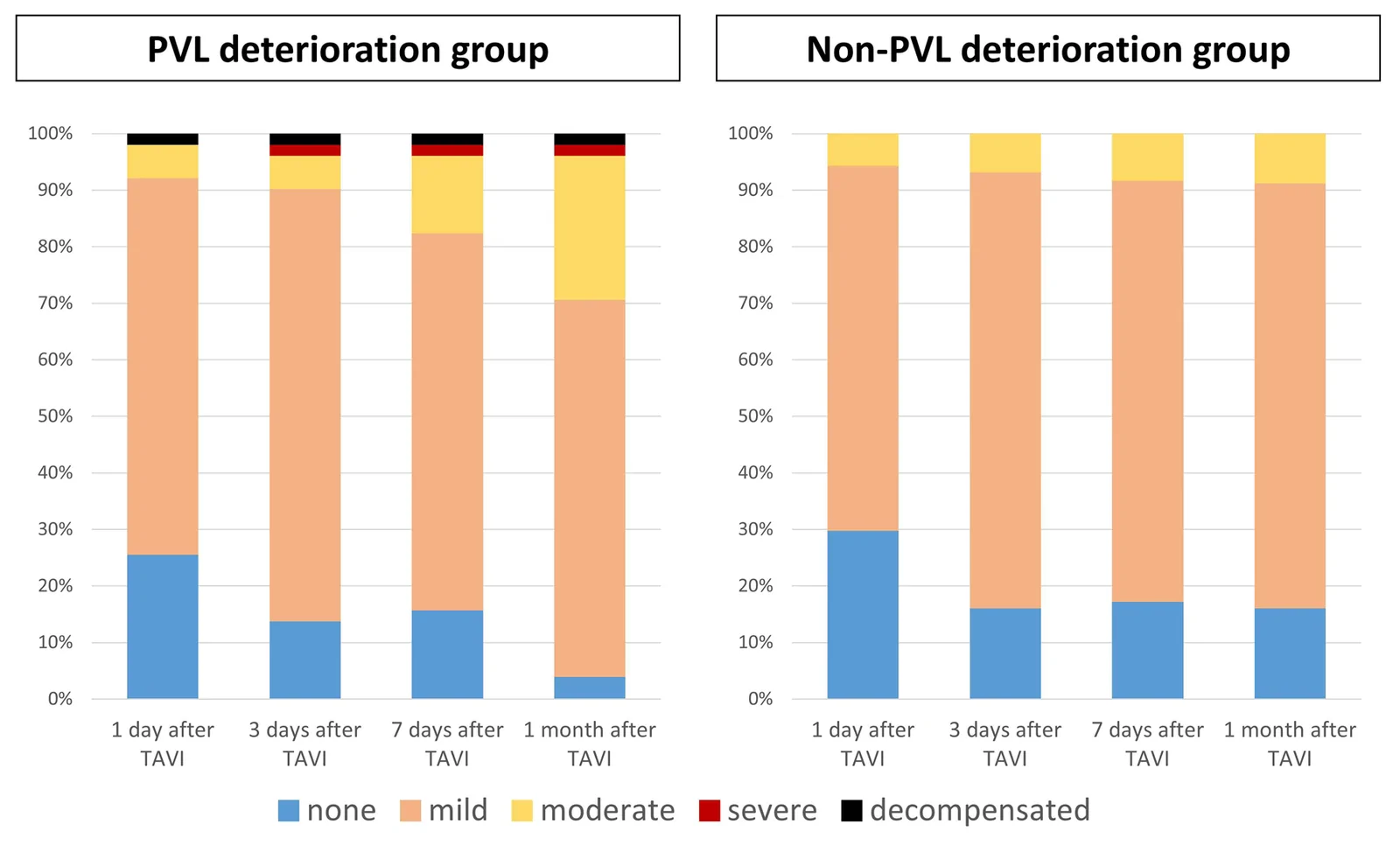
Severity of serum LDH elevation. LDH: lactate dehydrogenase; PVL: paravalvular leakage; TAVI: transcatheter aortic valve implantation.

### Temporal changes in PVL

Temporal changes in PVL are depicted in Figure 4. Among patients in the PVL deterioration group, 98% had none or trace PVL and 2% had mild PVL immediately after TAVI; at hospital discharge, 96.1% had mild PVL and 3.9% had mild-to-moderate PVL; at 1 month, 19.6% had none or trace PVL, 68.6% had mild PVL, 7.8% had mild-to-moderate PVL, and 3.9% had moderate PVL. Among patients in the non-PVL deterioration group, 84.4% had none or trace PVL, 14.9% had mild PVL, and 0.8% had mild-to-moderate PVL immediately after TAVI; at hospital discharge, 86.6% had none or trace PVL, 12.6% had mild PVL, and 0.8% had mild-to-moderate PVL; at 1 month, 86.3% had none or trace PVL, 11.8% had mild PVL, 1.5% had mild-to-moderate PVL, and 0.4% had moderate PVL.

**Figure 4:**
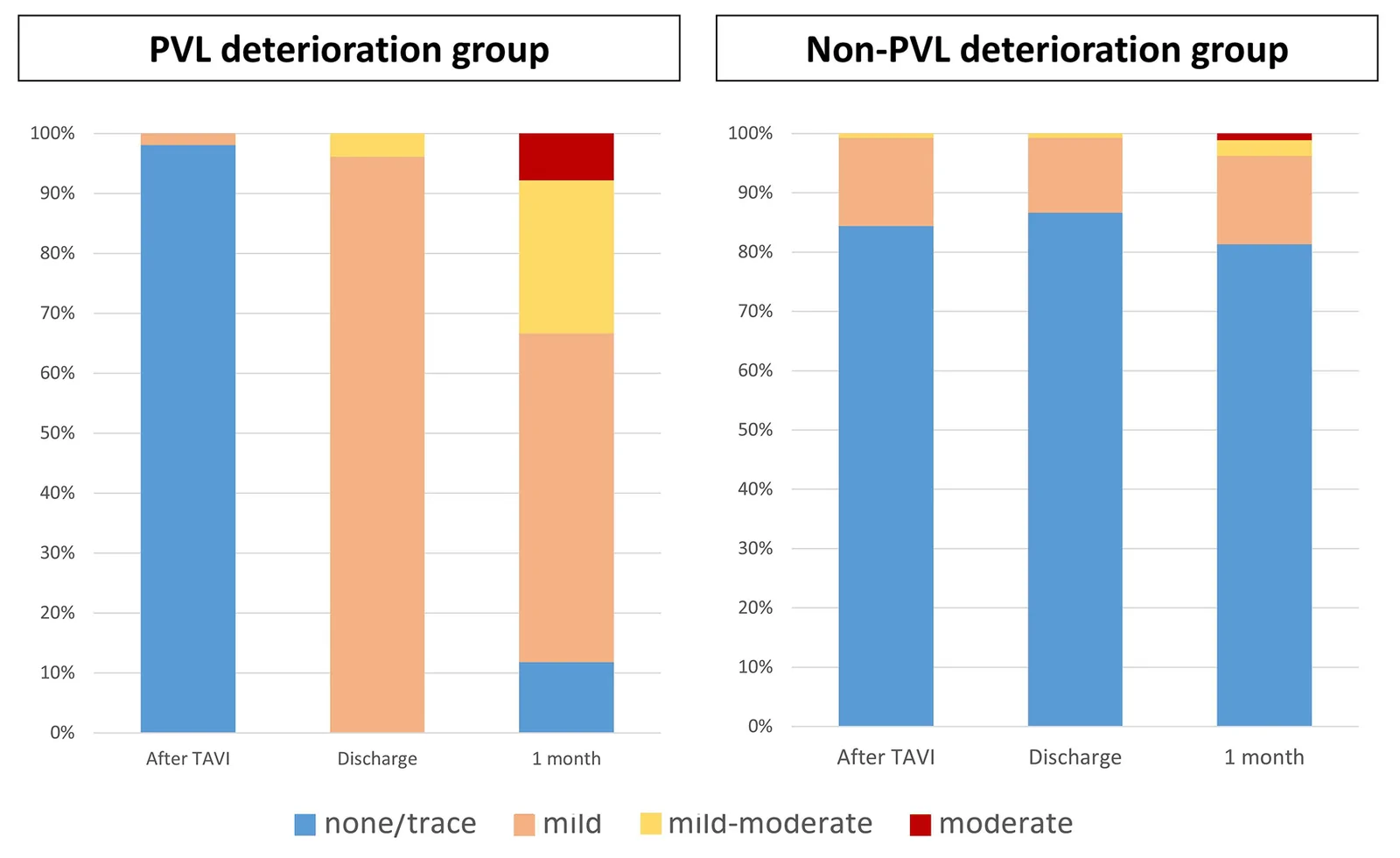
Changes in PVL severity after TAVI. PVL: paravalvular leakage; TAVI: transcatheter aortic valve implantation.

### Predictors of PVL deterioration

Figure 5 presents the ROC curve for the SI for predicting PVL deterioration. The SI demonstrated a sensitivity of 75.2% and specificity of 82.4%, with an area under the ROC curve of 0.84. The optimal cut-off value was 96.5% (OR: 13.6; 95% CI: 6.55–31.1; P < 0.01).

**Figure 5:**
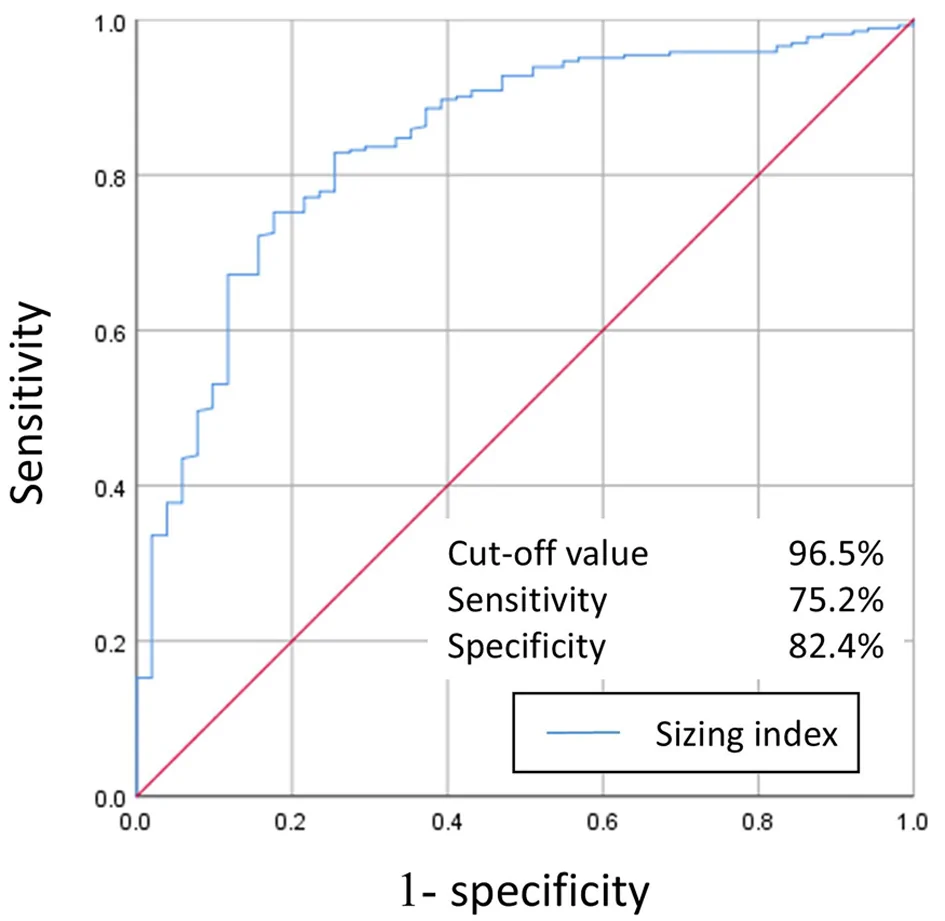
ROC curve for predicting paravalvular leakage deterioration using the sizing index. The area under the ROC curve was 0.84, with an optimal cut-off value of 96.5% (odds ratio: 13.6; 95% confidence interval: 6.55–31.1; P < 0.01). ROC: receiver operating characteristic.

Univariate and multivariate analyses of predictors of PVL deterioration are shown in Table 3. PVL deterioration occurred more frequently in patients with bicuspid than tricuspid aortic valve morphology (35.3% (6/17) vs. 15.2% (45/296)). In the univariable logistic regression analysis, bicuspid aortic valve (BAV) was associated with PVL deterioration (OR: 3.04; 95% CI: 1.08–8.55; P = 0.035). In a multivariate analysis, an aortic valve calcium score ≥ 1,435 (median) (OR: 6.54; 95% CI: 2.62–16.3; P < 0.01), use of S3UR (OR: 2.67; 95% CI: 1.19–6.01; P = 0.01), and an SI < 96.5 (OR: 10.5; 95% CI: 4.45–24.6; P < 0.01) were independent predictors of PVL deterioration in patients undergoing TAVI.

**Table 3:** Predictors of PVL Deterioration in Univariable and Multivariable Logistic Regression Analyses

	Univariate analysis		Multivariate analysis	
	OR (95% CI)	P value	OR (95% CI)	P value
AR ≥ mild	2.04 (1.09–4.02)	0.03	1.82 (0.81–4.07)	0.15
LVOT calcium ≥ moderate	3.05 (1.01–8.46)	0.048	2.48 (0.59–10.4)	0.21
Aortic valve calcium score ≥ 1,435 (median)	8.29 (3.82–20.8)	< 0.01	6.54 (2.62–16.3)	< 0.01
SAPIEN 3 Ultra RESILIA	4.44 (2.37–8.36)	< 0.01	2.67 (1.19–6.01)	0.01
Post-dilatation	0.16 (0.01–0.78)	0.02	0.41 (0.04–3.95)	0.44
Sizing index < 96.5	13.6 (6.55–31.1)	< 0.01	10.5 (4.45–24.6)	< 0.01
Bicuspid valve	3.04 (1.08–8.55)	0.035		

Values are mean ± standard deviation or n (%), unless otherwise specified. AR: aortic valve regurgitation; CI: confidence interval; LVOT: left ventricular outflow tract; OR: odds ratio; PVL: paravalvular leakage.

### Mortality according to the severity of serum LDH levels 1 month after TAVI

The median follow-up duration was 2.2 (IQR: 1.2–3.2) years. We conducted an exploratory analysis of all-cause mortality according to the severity of LDH elevation at 1 month after TAVI; Supplementary Material 5 (cr.elmerpub.com). There was no significant difference in the 3-year mortality between patients with moderate-or-greater serum LDH elevation and those with none-to-mild serum LDH elevation (43.9% vs. 34.7%; hazard ratio (HR): 1.25; 95% CI: 0.69–2.26; P = 0.46).

## Discussion

The principal findings of this study are threefold. First, the incidence of PVL deterioration at hospital discharge was 16%. Second, an SI < 96.5, aortic valve calcium score ≥ 1,435 (median), and use of S3UR were identified as independent predictors of PVL deterioration in patients undergoing TAVI. Third, serum LDH levels demonstrated an overall increasing trend up to 1 month after TAVI. Notably, LDH levels were significantly higher in patients with PVL deterioration than in those without, from post-procedural day 1 through 1-month follow-up.

### Incidence of PVL and deterioration

Post-procedural echocardiographic evaluation was routinely performed just before hospital discharge after TAVI, as the severity of PVL is associated with adverse clinical outcomes [11]. Previous studies have reported the incidence of mild PVL at hospital discharge, ranging from 7.9% to 43% with S3 valves and approximately 12.5% with S3UR valves [12–14]. In this study, the incidence of mild PVL at hospital discharge was 26.2%. This result is consistent with previous studies on S3 valves, but higher than previous studies on S3UR valves. The observed discrepancy may be attributable to the fact that S3 valves were used in approximately 75% of the patients. The observed PVL deterioration rate of 16.3% may reflect dynamic changes in valve behavior, including prosthetic valve recoil after deployment [3, 4]. However, studies comparing PVL severity immediately after TAVI and at hospital discharge remain limited. In this study, the median length of hospital stay was approximately 8 days, whereas a previous study documented it as short as 1 day [2]. Such a difference in post-procedural monitoring duration may have contributed to the higher rate of PVL deterioration in this study.

### Predictors of PVL deterioration after TAVI

Predictors of residual PVL after TAVI include left ventricular outflow tract eccentricity, discordant sizing, and three-leaflet calcification [15, 16]. However, predictors of short-term PVL deterioration after TAVI remain poorly understood. One proposed mechanism of PVL deterioration is prosthetic valve recoil [3, 4]. The elastic properties of the metallic frame used in transcatheter heart valves may lead to recoil after deployment. In particular, the cobalt-chromium alloy used in the frame of SAPIEN valves has been shown to influence the extent of recoil [17]. Additionally, during deployment of balloon-expandable prosthetic valves, local calcification and anatomical irregularities may impose excessive strain, stretching, and wall shear stress on the leaflet edges. This phenomenon may cause anatomical distortion at the interface between the prosthesis and the native annulus [18], which could potentially contribute to PVL deterioration. In this study, an SI < 96.5, an aortic valve calcium score ≥ 1,435, and use of the S3UR valve were independently associated with short-term PVL deterioration. Balloon-expandable valves are inherently susceptible to varying degrees of recoil. In cases with a lower SI, the potential space created by the recoil between the valve and the native annulus may lead to PVL deterioration. Similarly, a greater calcium burden may promote frame recoil. Sammour et al reported no significant relationship between the SI, the aortic valve calcium score, and valve recoil [4]. However, they evaluated recoil using fluoroscopic imaging rather than CT, which may be less accurate depending on the location of calcification. Their analyses were also limited to intraprocedural changes and post-procedural changes were not assessed, which may have affected the conclusions. Previous studies have calculated the SI based on the nominal device area, but they did not account for the actual balloon inflation volume (e.g., overfilling or underfilling), which may have influenced the outcomes [4, 18]. Although S3UR valves demonstrated improved hemodynamic performance compared to S3 valves, a recent study suggested their association with hemolysis [14]. One proposed mechanism of this involves increased shear stress between the textured polyethylene terephthalate outer skirt of S3UR valves and the native annulus [19]. To better elucidate these findings, larger cohort studies including both S3 and S3UR valves are warranted.

TAVI effectively stabilizes aortic stenosis and reduces the risk of future heart failure-related rehospitalizations. However, the presence of moderate or greater PVL after TAVI has been associated with both short- and long-term adverse outcomes [20, 21]. In this study, progression to mild or greater PVL occurred within a short time frame, even in patients with no or trace PVL immediately after the procedure. Although uncommon, hemolysis requiring clinical intervention—such as blood transfusion or balloon post-dilatation—may occur after TAVI [19, 22]. In this study, all patients with clinically significant hemolysis were in the PVL deterioration group, underscoring the importance of identifying and implementing preventive strategies to mitigate PVL deterioration and improve outcomes after TAVI. The parameters identified as predictors of PVL deterioration, may serve as valuable tools in making clinical decisions for patients with severe aortic stenosis undergoing TAVI.

In the present study, serial PVL assessment was performed using TTE, which is non-invasive and readily repeatable and therefore suitable for longitudinal follow-up. However, TTE may underestimate or incompletely characterize PVL because of limited acoustic windows, prosthetic valve-related acoustic shadowing, and eccentric or multiple regurgitant jets. TEE can provide more detailed anatomical assessment of PVL, particularly when TTE findings are inconclusive, but its semi-invasive nature limits its suitability for routine serial assessment.

Contemporary TAVI practice has increasingly shifted toward minimalist strategies and earlier discharge, with criteria-based fast-track pathways allowing selected patients to be safely discharged within 1–3 days after the procedure [23, 24]. In contrast, the relatively longer hospital stay in the present study allowed for serial assessment of PVL and laboratory parameters during the early post-procedural period and enabled us to capture dynamic changes in PVL before discharge. However, in the current era of fast-track TAVI, some of these changes may occur after hospital discharge. Therefore, early outpatient follow-up incorporating echocardiographic reassessment and, when clinically indicated, laboratory evaluation may be important for detecting evolving PVL after TAVI.

### Temporal changes in serum LDH and the incidence of clinically relevant hemolysis after TAVI

Hemolysis represents a notable complication of aortic valve interventions, occurring in 5–10% of surgical valve replacements using bioprosthetic valves and 9–28% of TAVI procedures [5, 25–28]. Serum LDH is often used as a surrogate marker to assess hemolysis severity. However, limited data are available on the temporal evolution of hemolysis after TAVI, and the post-operative trajectory remains incompletely characterized. In this study, we evaluated serial changes in serum LDH levels up to 1 month after the procedure. The findings of this study demonstrated a trend toward increasing LDH levels from immediately after the procedure through 1 month, with approximately 90% of patients exhibiting at least mild LDH elevation at 1 month follow-up. The underlying mechanism of LDH elevation after TAVI is thought to involve high shear stress and direct mechanical interactions between erythrocytes and adjacent structural components. Based on our observations, it is plausible that subclinical, mild LDH elevation—requiring no therapeutic intervention—occurs in most patients undergoing TAVI. In contrast, decompensated hemolytic anemia necessitating clinical intervention, including blood transfusion, appears to be exceedingly rare. Indeed, the reported incidence of clinically significant hemolysis after TAVI performed with balloon-expandable valves is approximately 1.7% [19]. In this study, clinically relevant hemolysis requiring treatment was observed in only 0.6% of patients, and the PVL deterioration group had a significantly greater increase in LDH levels and a higher incidence of moderate or greater LDH elevation after the procedure than the non-PVL deterioration group.

Higuchi et al reported similar findings, stating that, although the incidence of clinically significant hemolysis requiring intervention was low, more than half of the patients experienced PVL deterioration, with varying time points [22]. In this study, because TTE was performed just before hospital discharge, early changes in PVL were not adequately assessed; however, elevated LDH levels were already observed on post-procedural day 1 in the PVL deterioration group. This suggests that dynamic changes in PVL occur in the early post-procedural period, leading to increased shear stress and contributing to the progression of LDH elevation in this group.

Importantly, the association between early PVL deterioration and subsequent LDH elevation remained significant after adjustment for potential confounding factors. Furthermore, unlike LDH, CPK, AST, and ALT did not show parallel increases in patients with PVL deterioration. Nevertheless, LDH is a non-specific biomarker, and elevated LDH levels after TAVI may result from various conditions, including myocardial injury related to the procedure, hepatic injury or congestion, skeletal muscle injury, infection, inflammation, and malignancy. Although the absence of parallel increases in CPK, AST, and ALT suggests that the progressive LDH elevation associated with PVL deterioration may not be explained solely by non-specific procedural, skeletal muscle, or hepatic injury, our findings do not establish PVL-related hemolysis as the sole mechanism underlying LDH elevation.

From a clinical perspective, LDH levels alone cannot currently be used to guide management after TAVI. However, persistent or progressive LDH elevation may serve as an adjunctive marker prompting reassessment of PVL by echocardiography, particularly in patients with known residual PVL or early PVL deterioration. Future prospective, multicenter studies incorporating serial echocardiographic assessment and comprehensive laboratory evaluation of hemolysis are warranted to validate these findings. Further investigation integrating PVL morphology and anatomical characteristics with serial LDH changes may help identify patients at risk of clinically relevant PVL deterioration and associated LDH elevation and clarify their impact on long-term clinical outcomes after TAVI.

### Limitations

This study had five major limitations that need to be acknowledged. First, this was a retrospective single-center observational study with a relatively small study population and was not free from bias. Therefore, large-scale, multicenter studies are needed in the future. In addition, valve type was determined by the treatment period rather than by random allocation, with S3UR introduced after April 2023. Therefore, residual confounding related to the device era, including changes in procedural techniques and patient selection over time, cannot be excluded. Second, although several laboratory parameters were serially evaluated, comprehensive laboratory assessment specifically designed to diagnose hemolysis was not performed. Therefore, LDH elevation cannot be attributed exclusively to hemolysis, and the true incidence of subclinical hemolysis remains unknown. Third, prosthesis sizing relative to the native annulus was assessed according to the balloon inflation volume. Although this approach may offer a more clinically applicable evaluation, it deviates from the formula commonly used in previous studies—(nominal prosthesis area/CT annulus area − 1) × 100. Consequently, comparisons of prosthesis sizing with previous reports should be interpreted with caution. Fourth, PVL was serially assessed using TTE, and changes in a single PVL grade may have been influenced by loading conditions, acoustic windows, and measurement variability. Therefore, some degree of misclassification of PVL deterioration cannot be excluded. Lastly, this study did not evaluate the subsequent clinical course of patients who experienced short-term PVL deterioration. Therefore, the mid- to long-term impact of post-TAVI PVL deterioration remains unclear. Further large-scale studies are warranted to address this important issue.

### Conclusions

Early PVL deterioration after TAVI with a balloon-expandable valve may be associated with the development of serum LDH elevation. Notably, a low sizing ratio, severe aortic valve calcification, and use of the S3UR prosthesis were associated with an increased likelihood of PVL deterioration. These findings underscore the importance of selecting a prosthetic valve with an adequate sizing ratio to minimize PVL deterioration after TAVI.

## Supplementary Material

Suppl 1 Baseline patient characteristics.

Suppl 2 Serial percent changes in blood tests through TAVI treatment. TAVI: transcatheter aortic valve implantation.

Suppl 3 Serial changes in blood tests through TAVI treatment. TAVI: transcatheter aortic valve implantation.

Suppl 4 Multivariable linear regression analyses for LDH at 1 Month After TAVI. Primary analysis: LDH levels at 1 month. Sensitivity analysis: percent change in LDH from baseline to 1 month. LDH: lactate dehydrogenase; TAVI: transcatheter aortic valve implantation.

Suppl 5 Kaplan–Meier estimates of all-cause mortality according to LDH elevation at 1 month after TAVI. LDH: lactate dehydrogenase; TAVI: transcatheter aortic valve implantation; HR: hazard ratio; CI: confidence interval.

## Data Availability

The datasets used and/or analyzed during the current study are available from the corresponding author on reasonable request.

## Abbreviations

ALP: alkaline phosphatase
ALT: alanine aminotransferase
AST: aspartate aminotransferase
AUC: area under the curve
CI: confidence interval
CPK: creatine phosphokinase
CT: computed tomography
HR: hazard ratio
IQR: interquartile range
LDH: lactate dehydrogenase
OR: odds ratio
PPM: prosthesis–patient mismatch
PVL: paravalvular leakage
ROC: receiver operating characteristic
S3: SAPIEN 3
S3UR: SAPIEN 3 Ultra RESILIA
SI: sizing index
ST: standard deviation
TAVI: transcatheter aortic valve implantation
T-Bil: total bilirubin
TTE: transthoracic echocardiography
UA: uric acid
VARC-3: Valve Academic Research Consortium-3
