# Supplementary material for: Association Between Changes in Paravalvular Leakage After Transcatheter Aortic Valve Implantation and Serum Lactate Dehydrogenase
Source: Cardiol Res. 2026 Oct 24;17(6):630–41. doi: 10.14740/cr2307 (PMC13724139; doi:10.14740/cr2307)
Supplement: Supplementary file 1 [file cr-17-06-630-s001.docx]

**Suppl 1.** Baseline Patient Characteristics

|  | Overall | PVL deterioration group | Non-PVL deterioration group | *P* value |
| --- | --- | --- | --- | --- |
| Number of Patients | 313 | 51 | 262 |  |
| Height, cm | 153.1 ± 9.7 | 153.0 ± 9.3 | 153.1 ± 9.8 | 0.95 |
| Weight, kg | 53.3 ± 10.5 | 55.0 ±10.5 | 53.1 ±10.5 | 0.23 |
| BMI, kg/m^2^ | 22.7 ± 3.5 | 23.4 ± 3.1 | 22.6 ± 3.6 | 0.14 |
| Clinical frailty scale |  |  |  | 0.25 |
| ≤3 | 199 (63.6) | 36 (70.6) | 163 (62.2) |  |
| ≥4 | 114 (36.4) | 15 (29.4) | 99 (37.8) |  |
| Kats index |  |  |  | 0.11 |
| ≥5 | 294 (93.6) | 50 (98.0) | 244 (92.8) |  |
| ≤4 | 20 (6.4) | 1 (2.0) | 19 (7.2) |  |
| Society of Thoracic Surgeons risk score | 7.4 ± 5.5 | 7.0 ± 4.3 | 7.4 ± 5.7 | 0.59 |
| Previous PCI | 54 (17.3) | 11 (21.6) | 43 (16.4) | 0.38 |
| Previous cardiac surgery | 6 (1.9) | 1 (2.0) | 5 (1.9) | 0.98 |
| Atrial fibrillation | 29 (9.3) | 3 (5.9) | 26 (9.9) | 0.34 |
| Permanent pacemaker | 16 (5.1) | 5 (9.8) | 11 (4.2) | 0.13 |
| Echocardiographic parameters |  |  |  |  |
| LVDd, mm | 43.1 ± 6.1 | 42.3 ± 4.9 | 43.3 ± 6.3 | 0.28 |
| LVDs, mm | 28.5 ± 7.5 | 27.0 ± 5.6 | 28.8 ± 7.8 | 0.11 |
| Computed tomography parameters |  |  |  |  |
| Annulus short axis, mm | 21.2 ± 2.0 | 20.6 ± 1.9 | 21.3 ± 2.0 | 0.02 |
| Annulus long axis, mm | 26.4 ± 2.7 | 26.0 ± 2.5 | 26.4 ± 2.8 | 0.34 |
| Annulus long/short axis | 1.25 ± 0.1 | 1.27 ± 0.09 | 1.24 ± 0.1 | 0.09 |
| Valsalva area, mm^2^ | 803 ± 179 | 814 ± 200 | 801 ± 176 | 0.64 |
| Valsalva perimeter, mm | 104.5 ± 12.2 | 105.7 ± 13.9 | 104.3 ± 11.9 | 0.46 |
| STJ area, mm^2^ | 609 ± 140 | 625 ± 139 | 606 ± 140 | 0.36 |
| STJ perimeter, mm | 86.8 ± 9.8 | 87.8 ± 9.7 | 86.7 ± 9.8 | 0.44 |
| Medication use |  |  |  |  |
| Aspirin | 206 (65.8) | 33 (64.7) | 173 (66.0) | 0.86 |
| Thienopyridine | 46 (14.7) | 12 (23.5) | 34 (13.0) | 0.06 |
| Anticoagulant | 77 (24.6) | 14 (27.5) | 63 (24.1) | 0.61 |
| β-blocker | 89 (28.4) | 12 (23.5) | 77 (29.4) | 0.39 |
| ACE-I/ARB | 148 (47.3) | 25 (49.0) | 123 (47.0) | 0.79 |

Values are mean ± standard deviation or n (%), unless otherwise specified.

ACE-I: angiotensin-converting enzyme inhibitor, ARB: angiotensin receptor blocker, BMI: body mass index, LVDd: left ventricular end-diastolic diameter, LVDs: left ventricular end-systolic diameter, PCI: percutaneous coronary intervention, STJ: sino-tubular junction.
