# Supplementary material for: Association Between Changes in Paravalvular Leakage After Transcatheter Aortic Valve Implantation and Serum Lactate Dehydrogenase
Source: Cardiol Res. 2026 Oct 24;17(6):630–41. doi: 10.14740/cr2307 (PMC13724139; doi:10.14740/cr2307)
Supplement: Supplementary file 2 [file cr-17-06-630-s002.docx]

**Suppl 2.** Serial Percent Changes in Blood Tests Through TAVI Treatment

|  | Overall | PVL deterioration group | Non-PVL deterioration group | *P* value |
| --- | --- | --- | --- | --- |
| Number of patients | 313 | 51 | 262 |  |
| Percent changes in LDH |  |  |  |  |
| At 1 day, % | 26.1 ± 40.0 | 38.2 ± 69.5 | 23.8 ± 30.9 | 0.02 |
| At 3 days, % | 37.3 ± 45.0 | 58.8 ± 80.0 | 33.1 ± 33.0 | <0.01 |
| At 7 days, % | 40.7 ± 49.9 | 65.4 ± 84.4 | 36.0 ± 38.4 | <0.01 |
| At 1 month, % | 48.0 ± 52.9 | 94.3 ± 93.0 | 38.1 ± 32.0 | <0.01 |
| Percent changes in ALP |  |  |  |  |
| At 1 day, % | −1.0 ± 16.4 | −2.6 ± 12.3 | −0.6 ± 17.0 | 0.43 |
| At 3 days, % | 3.2 ± 17.3 | 4.3 ± 13.4 | 3.0 ± 17.9 | 0.65 |
| At 7 days, % | 8.8 ± 22.7 | 7.8 ± 19.5 | 9.0 ± 23.3 | 0.72 |
| At 1 month, % | 29.3 ± 29.4 | 24.2 ± 23.8 | 30.3 ± 30.4 | 0.18 |
| Percent changes in ALT |  |  |  |  |
| At 1 day, % | −11.8 ± 38.0 | −7.7 ± 33.7 | −12.6 ± 38.8 | 0.4 |
| At 3 days, % | −18.6 ± 38.0 | −10.6 ± 31.6 | −20.1 ± 39.0 | 0.1 |
| At 7 days, % | −3.7 ± 60.6 | −0.5 ± 38.3 | −4.4 ± 64.0 | 0.64 |
| At 1 month, % | 14.5 ± 47.8 | 15.6 ± 46.4 | 14.3 ± 48.1 | 0.86 |
| Percent changes in AST |  |  |  |  |
| At 1 day, % | 14.8 ± 44.7 | 16.2 ± 28.5 | 14.6 ± 47.3 | 0.81 |
| At 3 days, % | 9.1 ± 47.9 | 10.6 ± 33.3 | 8.8 ± 50.2 | 0.81 |
| At 7 days, % | 3.8 ± 36.1 | 7.6 ± 37.5 | 3.1 ± 35.8 | 0.42 |
| At 1 month, % | 6.6 ± 38.9 | 13.6 ± 42.1 | 5.1 ± 37.8 | 0.15 |
| Percent changes in hemoglobin |  |  |  |  |
| At 1 day, % | −0.8 ± 11.2 | −0.1 ± 10.2 | −0.1 ± 11.4 | 0.59 |
| At 3 days, % | 0.3 ± 11.3 | 2.3 ± 10.9 | −0.1 ± 11.3 | 0.15 |
| At 7 days, % | −0.3 ± 11.6 | 0.8 ± 11.8 | −0.5 ± 11.5 | 0.48 |
| At 1 month, % | 6.5 ± 13.0 | 4.5 ± 12.5 | 6.9 ± 13.1 | 0.24 |
| Percent changes in CPK |  |  |  |  |
| At 1 day, % | 47.0 ± 88.7 | 32.8 ± 65.9 | 49.7 ± 92.4 | 0.39 |
| At 3 days, % | 36.7 ± 75.3 | 20.3 ± 55.1 | 39.8 ± 78.4 | 0.23 |
| At 7 days, % | 18.8 ± 72.0 | 6.7 ± 45.1 | 21.1 ± 76.0 | 0.59 |
| At 1 month, % | 9.1 ± 74.1 | 0.2 ± 35.6 | 11.1 ± 80.0 | 0.83 |
| Percent changes in T-Bil |  |  |  |  |
| At 1 day, % | 34.7 ± 44.0 | 47.6 ± 44.8 | 32.2 ± 43.5 | 0.02 |
| At 3 days, % | 32.9 ± 47.7 | 45.2 ± 52.0 | 30.5 ± 46.6 | 0.04 |
| At 7 days, % | 19.9 ± 46.7 | 31.6 ± 52.0 | 17.6 ± 45.3 | 0.051 |
| At 1 month, % | 6.8 ± 43.6 | 16.5 ± 43.9 | 4.8 ± 43.4 | 0.09 |
| Percent changes in UA |  |  |  |  |
| At 1 day, % | −1.9 ± 15.1 | −1.9 ± 12.8 | −1.8 ± 15.6 | 0.99 |
| At 3 days, % | −4.0 ± 22.0 | −2.2 ± 19.7 | −4.3 ± 22.4 | 0.54 |
| At 7 days, % | −0.2 ± 26.3 | −3.2 ± 22.5 | 0.4 ± 27.0 | 0.38 |
| At 1 month, % | 3.3 ± 24.3 | 2.3 ± 18.8 | 3.5 ± 25.4 | 0.75 |
| Percent changes in creatinine |  |  |  |  |
| At 1 day, % | −1.7 ± 15.2 | −3.2 ± 22.8 | −1.4 ± 13.1 | 0.47 |
| At 3 days, % | 0 ± 16.9 | 1.4 ± 25.7 | −0.2 ± 14.4 | 0.57 |
| At 7 days, % | −1.0 ± 16.5 | 0.1 ± 25.8 | −1.2 ± 13.7 | 0.62 |
| At 1 month, % | 7.5 ± 20.6 | 8.9 ± 29.4 | 7.1 ± 18.1 | 0.6 |

Values are mean ± standard deviation or n (%), unless otherwise specified.

ALP: alkaline phosphatase, ALT: alanine aminotransferase, AST: aspartate aminotransferase, CPK: creatine phosphokinase, LDH: lactate dehydrogenase, PVL: paravalvular leakage, TAVI: transcatheter aortic valve implantation, T-Bil: total bilirubin, UA: uric acid.
