# Supplementary material for: Association Between Changes in Paravalvular Leakage After Transcatheter Aortic Valve Implantation and Serum Lactate Dehydrogenase
Source: Cardiol Res. 2026 Oct 24;17(6):630–41. doi: 10.14740/cr2307 (PMC13724139; doi:10.14740/cr2307)
Supplement: Supplementary file 3 [file cr-17-06-630-s003.docx]

**Suppl 3.** Serial Changes in Blood Tests Through TAVI Treatment

|  | Overall | PVL deterioration group | Non-PVL deterioration group | *P* value |
| --- | --- | --- | --- | --- |
| Number of patients | 313 | 51 | 262 |  |
| Baseline |  |  |  |  |
| LDH, U/L | 210.4 ± 51.0 | 208.2 ± 40.9 | 210.8 ± 52.8 | 0.74 |
| ALP, U/L | 68.4 ± 28.3 | 67.7 ± 37.6 | 68.5 ± 26.1 | 0.85 |
| ALT, U/L | 14.2 ± 12.3 | 14.8 ± 11.3 | 14.1 ± 12.5 | 0.71 |
| AST, U/L | 23.8 ± 15.0 | 25.0 ± 17.7 | 23.6 ± 14.4 | 0.56 |
| Hemoglobin, g/dL | 10.4 ± 1.6 | 10.6 ± 1.7 | 10.4 ± 1.5 | 0.32 |
| CPK, U/L | 91.1 ± 56.0 | 101.8 ± 61.4 | 89.0 ± 54.7 | 0.25 |
| T-Bil, mg/dL | 0.5 ± 0.3 | 0.6 ± 0.3 | 0.5 ± 0.2 | 0.17 |
| UA, mg/dL | 5.3 ± 1.6 | 5.7 ± 1.9 | 5.2 ± 1.5 | 0.03 |
| Creatinine, mg/dL | 1.8 ± 1.9 | 1.5 ± 1.3 | 1.8 ± 2.0 | 0.25 |
| At 1 day |  |  |  |  |
| LDH, U/L | 259.9 ± 87.1 | 281.8 ± 143.4 | 255.6 ± 70.8 | 0.049 |
| ALP, U/L | 67.2 ± 30.4 | 65.2 ± 35.4 | 67.6 ± 29.4 | 0.6 |
| ALT, U/L | 12.1 ± 12.6 | 12.7 ± 8.2 | 11.9 ± 13.3 | 0.69 |
| AST, U/L | 26.2 ± 16.5 | 27.7 ± 15.2 | 25.9 ± 16.8 | 0.48 |
| Hemoglobin, g/dL | 10.3 ± 1.6 | 10.6 ± 1.9 | 10.2 ± 1.5 | 0.11 |
| CPK, U/L | 111.3 ± 67.0 | 116.1 ± 67.8 | 110.3 ± 67.8 | 0.46 |
| T-Bil, mg/dL | 0.7 ± 0.4 | 0.8 ± 0.6 | 0.7 ± 0.3 | <0.01 |
| UA, mg/dL | 5.1 ± 1.6 | 5.6 ± 1.9 | 5.1 ± 1.5 | 0.03 |
| Creatinine, mg/dL | 1.8 ± 2.1 | 1.5 ± 1.4 | 1.9 ± 2.2 | 0.2 |
| At 3 days |  |  |  |  |
| LDH, U/L | 282.2 ± 95.5 | 322.9 ± 163.7 | 268.2 ± 80.2 | <0.01 |
| ALP, U/L | 69.6 ± 30.1 | 69.1 ± 36.6 | 69.7 ± 28.8 | 0.89 |
| ALT, U/L | 11.1 ± 10.4 | 11.9 ± 6.4 | 10.9 ± 11.0 | 0.52 |
| AST, U/L | 24.1 ± 11.9 | 25.2 ± 9.2 | 24.0 ± 12.4 | 0.51 |
| Hemoglobin, g/dL | 10.4 ± 1.6 | 10.9 ± 2.0 | 10.3 ± 1.5 | 0.02 |
| CPK, U/L | 99.6 ± 48.1 | 99.9 ± 40.5 | 99.5 ± 49.5 | 0.56 |
| T-Bil, mg/dL | 0.7 ± 0.4 | 0.8 ± 0.5 | 0.7 ± 0.3 | 0.01 |
| UA, mg/dL | 5.0 ± 1.6 | 5.5 ± 1.8 | 4.9 ± 1.5 | 0.01 |
| At 7 days |  |  |  |  |
| LDH, U/L | 289.1 ± 101.4 | 335.1 ± 167.5 | 280.1 ± 80.2 | <0.01 |
| ALP, U/L | 72.9 ± 31.7 | 70.2 ± 33.9 | 73.4 ± 31.2 | 0.5 |
| ALT, U/L | 13.1 ± 12.8 | 13.3 ± 7.0 | 13.0 ± 13.7 | 0.91 |
| AST, U/L | 24.5 ± 13.7 | 23.8 ± 10.0 | 24.6 ± 14.3 | 0.7 |
| Hemoglobin, g/dL | 10.4 ± 1.6 | 10.7 ± 1.9 | 10.3 ± 1.6 | 0.11 |
| CPK, U/L | 89.3 ± 53.9 | 96.6 ± 54.4 | 87.8 ± 53.8 | 0.20 |
| T-Bil, mg/dL | 0.6 ± 0.3 | 0.7 ± 0.4 | 0.6 ± 0.3 | <0.01 |
| UA, mg/dL | 5.1 ± 1.6 | 5.5 ± 1.9 | 5.1 ± 1.5 | 0.12 |
| Creatinine, mg/dL | 2.0 ± 2.5 | 1.7 ± 1.9 | 2.1 ± 2.5 | 0.3 |
| At 1 month |  |  |  |  |
| LDH, U/L | 306.8 ± 138.0 | 404.2 ± 268.0 | 285.9 ± 74.6 | <0.01 |
| ALP, U/L | 85.2 ± 37.2 | 81.2 ± 36.6 | 86.0 ± 37.4 | 0.41 |
| ALT, U/L | 14.8 ± 12.3 | 14.4 ± 6.1 | 14.8 ± 13.2 | 0.82 |
| AST, U/L | 23.4 ± 11.0 | 24.9 ± 8.7 | 23.1 ± 11.4 | 0.29 |
| Hemoglobin, g/dL | 11.1 ± 1.6 | 11.1 ± 1.8 | 11.1 ± 1.5 | 0.91 |
| CPK, U/L | 85.9 ± 48.9 | 93.7 ± 47.0 | 84.2 ± 49.3 | 0.08 |
| T-Bil, mg/dL | 0.5 ± 0.3 | 0.6 ± 0.3 | 0.5 ± 0.2 | <0.01 |
| UA, mg/dL | 5.3 ± 1.6 | 5.6 ± 1.7 | 5.3 ± 1.5 | 0.12 |
| Creatinine, mg/dL | 1.8 ± 2.0 | 1.7 ± 1.9 | 1.8 ± 2.1 | 0.83 |

Values are mean ± standard deviation or n (%), unless otherwise specified.

ALP: alkaline phosphatase, ALT: alanine aminotransferase, AST: aspartate aminotransferase, CPK: creatine phosphokinase, LDH: lactate dehydrogenase, PVL: paravalvular leakage, TAVI: transcatheter aortic valve implantation, T-Bil: total bilirubin, UA: uric acid.
