# Supplementary material for: Association Between Changes in Paravalvular Leakage After Transcatheter Aortic Valve Implantation and Serum Lactate Dehydrogenase
Source: Cardiol Res. 2026 Oct 24;17(6):630–41. doi: 10.14740/cr2307 (PMC13724139; doi:10.14740/cr2307)
Supplement: Supplementary file 4 [file cr-17-06-630-s004.docx]

**Suppl4.** Multivariable Linear Regression Analyses for LDH at 1 Month After TAVI

A. Primary analysis: LDH levels at 1 month

| Variables | β coefficient | 95% CI | *P* value |
| --- | --- | --- | --- |
| PVL deterioration | 124.1 | 47.1–201.1 | 0.002 |
| Baseline LDH, per 1 U/L | 1.0 | 0.28–1.71 | 0.006 |
| Age, per 1 year | 0.95 | – 2.17–4.07 | 0.549 |
| Male sex | 17.4 | – 26.7–61.6 | 0.439 |
| Baseline serum creatinine, per 1 mg/dL | – 0.18 | – 5.61–5.25 | 0.948 |

Dependent variable: LDH level at 1 month (U/L), Adjusted R^2^ = 0.219

B. Sensitivity analysis: Percent change in LDH from baseline to 1 month

| Variables | β coefficient | 95% CI | *P* value |
| --- | --- | --- | --- |
| PVL deterioration | 56.3 | 29.7–82.8 | <0.001 |
| Age, per 1 year | – 0.06 | – 1.25–1.13 | 0.922 |
| Male sex | 7.41 | – 5.64–20.45 | 0.266 |
| Baseline creatinine, per 1 mg/dL | – 0.47 | – 3.10–2.16 | 0.726 |

Dependent variable: Percent change in LDH from baseline to 1 month (%), Adjusted R^2^ = 0.157

β coefficients represent the adjusted difference in the dependent variable associated with each covariate. PVL deterioration was defined as an increase of at least one PVL grade from the immediate postprocedural assessment to the predischarge assessment. Heteroscedasticity-robust standard errors were used. CI, confidence interval; LDH, lactate dehydrogenase; PVL, paravalvular leakage; TAVI, transcatheter aortic valve implantation.
