# Supplementary material for: Association Between Changes in Paravalvular Leakage After Transcatheter Aortic Valve Implantation and Serum Lactate Dehydrogenase
Source: Cardiol Res. 2026 Oct 24;17(6):630–41. doi: 10.14740/cr2307 (PMC13724139; doi:10.14740/cr2307)
Supplement: Supplementary file 5 [file cr-17-06-630-s005.docx]

**Suppl 5.** Kaplan–Meier Estimates of All-Cause Mortality According to LDH Elevation at 1 Month After TAVI. LDH: lactate dehydrogenase, TAVI: transcatheter aortic valve implantation.
